# Early Clinical Response of a Large Amebic Liver Abscess to Eravacycline Prior to Metronidazole Initiation: A Case Report

**DOI:** 10.1155/crdi/1368034

**Published:** 2026-02-09

**Authors:** Jamal A. Anthony, Andrew Robinson, Lisa Pedroza

**Affiliations:** ^1^ Department of Infectious Diseases, Cooper University Hospital, Camden, 08103, New Jersey, USA, cooperhealth.org; ^2^ Cooper Medical School of Rowan University, Camden, 08103, New Jersey, USA, rowan.edu; ^3^ Department of Internal Medicine, Cooper University Hospital, Camden, 08103, New Jersey, USA, cooperhealth.org

## Abstract

*Entamoeba histolytica* is an amebic protozoan that is often asymptomatic, with many patients being asymptomatic carriers. However, 10 percent of cases manifest with amebic dysentery and, less commonly, amebic liver abscesses. Amebic liver abscesses are the most common extraintestinal manifestation of *E. histolytica*. Patients may remain asymptomatic for years before presenting with active infection. Treatment of *E. histolytica* amebic abscesses with nitroimidazoles, such as metronidazole and tinidazole, is a long‐established approach that has remained unchanged since its discovery in the 1960s. However, given that this condition is known to be fatal without treatment, the outcome for a patient who cannot tolerate nitroimidazole therapy can be catastrophic. We present the case of a 54‐year‐old male who presented with what was initially thought to be a large, pyogenic liver abscess and who was started on standard empiric antibiotic therapy and percutaneous drainage. However, due to a lack of clinical response, his regimen was changed to eravacycline, a novel halogenated tetracycline, which led to a good response. Interestingly, the abscess was later discovered to be amebic in this patient, who had a low‐risk history for *E. histolytica* infection. This case demonstrates a clinical response to eravacycline monotherapy in a patient with *E. histolytica* liver abscess. We re‐examine the history of tetracyclines in the treatment of parasitic infections and consider the new, more potent halogenated tetracyclines as a potential alternative to the current standard of care.

## 1. Introduction

*Entamoeba* *histolytica* is the third leading cause of death from parasitic infections [1]. Although 90% of *E. histolytica* infections are asymptomatic, in endemic regions, there have been reports of nearly 50 million people becoming symptomatic, with up to 100,000 deaths yearly [1–3]. Individuals colonized with Entamoeba mostly carry either *E. histolytica* or *E. dispar*; however, *E. histolytica* is the pathogenic species that causes amebic colitis and extraintestinal amebiasis. *E. dispar* is nonpathogenic and is usually without clinical significance [1, 3].

Risk factors for infection are mostly related to fecal–oral transmission from poor hand hygiene, defecation into water sources such as rivers, and proximity to animals [1, 2]. As a result, infections occur with a higher prevalence in countries with low socioeconomic status and poor public health practices. Countries with high infection rates include India, Africa, Mexico, and Central and South America [1]. Additionally, there are reports of increased risk of transmission from homosexual or bisexual males due to the risk of fecal–oral contamination through oral and anal sex [1, 3].

While the infection rate is higher in developing countries, it is significant in developed countries as well, due to higher immigration rates and the sometimes decade‐long latency period from infection to symptom onset. In developed countries, infection is seen in individuals exposed to endemic areas, such as immigrants or travelers [1–3]. Amebic colitis generally affects males and females of all ages equally, albeit there is a predilection for amebic liver abscesses (ALAs) in males aged 18 to 50 [1].

*E. histolytica* can range from asymptomatic to severely symptomatic, ranging from intermittent bloating, diarrhea, and flatulence to overt dysentery and fulminant colitis with toxic megacolon [3]. Of the 10% of patients who do become symptomatic, they may also have extraintestinal manifestations, the most common of which is ALA, as presented in this case. Signs of ALA include abdominal pain, nausea, anorexia, weight loss, and fever [1–3]. Untreated ALAs have been associated with rupture, septic emboli, and, with larger abscesses, portal vein and hepatic vein thrombosis complicated by ischemic hepatitis [2]. The potential for high morbidity and mortality highlights the importance of treatment, even in asymptomatic carriers who can continue to transmit protozoa in their environment.

We present the case of an immunocompetent male patient with a large liver abscess secondary to *E. histolytica* and detail the unique aspect of his treatment course that led to clinical improvement.

## 2. Case Description

A middle‐aged male with a past medical history of childhood rheumatic fever presented to an outside hospital (OSH) with fever, abdominal pain, and jaundice. He reported being in his usual state of health until 2 weeks prior, when he started experiencing intermittent right flank pain, which he initially attributed to exercise. He then developed associated intermittent fevers and jaundice, leading him to seek medical attention.

**FIGURE 1:**
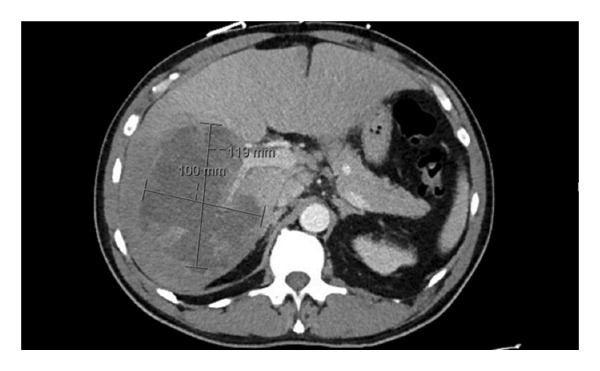
Still image of large complex liver abscess of the right lobe.

At the OSH, laboratory results showed an initial leukocytosis (17.2 × 10^3^/L; NR: 4.5–11.0 × 10^3^/L), direct hyperbilirubinemia, and transaminasemia (See Table 1). A computed tomography (CT) of the abdomen and pelvis revealed a large hypoattenuating mass in the right hepatic lobe (see Figure 1). He was started empirically on vancomycin and ampicillin–sulbactam given concerns for pyogenic liver abscess and was transferred to our tertiary care facility for interventional radiology (IR)–guided drainage.

**TABLE 1:** Laboratory findings showing significant transaminasemia and direct hyperbilirubinemia, which gradually improved with drainage and appropriate antibiotic therapy.

**Liver function test result throughout admission**																										
**Admission**																										**Discharge**
**Admission day**	**Day 1**	**Day 2**	**Day 3**	**Day 4**	**Day 5**	**Day 6**	**Day 7**	**Day 8**	**Day 9**	**Day 10**	**Day 10**	**Day 11**	**Day 12**	**Day 13**	**Day 13**	**Day 14**	**Day 15**	**Day 16**	**Day 17**	**Day 18**	**Day 19**	**Day 20**	**Day 21**	**Day 22**	**Day 23**	**Day 24**

AST (NR: 0–40 IU/L)	717	539	1064	645	513	357	421	192	187	126	129	117	97	91	76	59	62	63	65	56	49	43	45	44	39	57
ALT (NR: 0–44 IU/L)	695	639	783	629	560	473	434	301	269	218	203	165	139	134	117	104	91	87	78	72	68	58	50	47	37	34
Alkaline phosphatase (NR: 44–121 IU/L)	202	222	227	265	250	267	272	227	277	262	284	307	290	330	330	313	304	315	351	344	358	470	500	611	593	723
Bilirubin total (NR: 0–1.2 mg/dL)	1.7	1.8	3.0	3.3	4.5	4.8	4.6	4.1	4.4	4.6	3.6	4.4	4.5	3.4	2.4	2.3	2.2	1.9	1.8	1.5	1.6	1.3	1.1	1.2	1.0	0.9
Bilirubin direct (NR: 0–0.3 mg/dL)	1.2	1.3	2.4	2.7	3.9	4.4	3.9	3.3	3.9	4.2	3.2	3.5	4.0	2.8	1.9	1.7	1.4	1.3	1.2	0.9	1.0	0.8	0.6	0.8	0.6	0.4

Upon arrival, the patient was febrile (100.7°F) and mildly tachycardic (108 beats per minute). New laboratory results indicated improving leukocytosis and worsening transaminasemia and hyperbilirubinemia (see Table 1). His antibiotics were changed to vancomycin and piperacillin–tazobactam.

**FIGURE 2:**
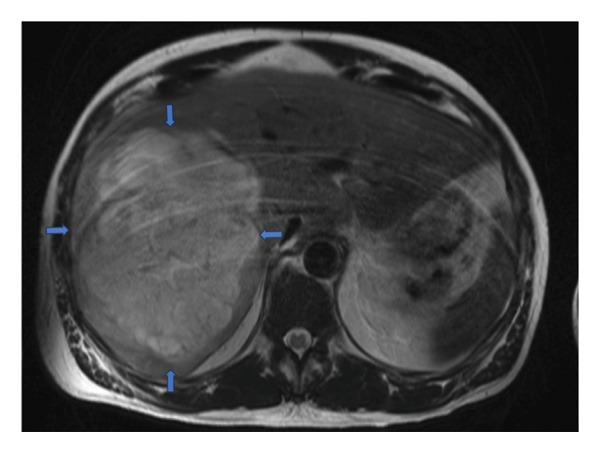
MRCP imaging showing diffusely enlarged liver with a heterogenous, T2 hyperintense, and T1 hypointense cystic lesion involving almost the entire right lobe of the liver measures approximately 15.3 × 12.5 × 19.5 cm (AP × TV × CC) with peripheral enhancement and peripheral incomplete septations in addition to mildly dilated right intrahepatic bile ducts from local mass effect. Blue arrows mark the borders.

Further history revealed pale stools and unintentional weight loss of 20 lbs over the preceding week. He denied a family or personal history of malignancy. Given this information, there was concern that this liver lesion could potentially be a necrotic malignant mass. As such, a magnetic resonance cholangiopancreatography (MRCP) was performed to evaluate the lesion before IR drainage. The results were consistent with a hepatic abscess, with no malignancy detected (see Figure 2).

His social history indicated that he grew up near a farm in the Caribbean, moved to the United States at age 18, and last visited the Caribbean two years prior. He denied any other recent travel, raw food consumption, and substance use. He had never been tested for HIV and was in a monogamous relationship with his wife.

On Day 4, the patient underwent IR drainage with drain placement, yielding thick purulent discharge. Bacterial, fungal, and acid–fast bacilli (AFB) cultures were sent. Despite receiving antibiotics, he continued to experience high‐grade fevers, albeit he was otherwise hemodynamically stable. On Day 6, the drain was upsized, and 1 L of discharge was aspirated. Since prior aspirate cultures showed no growth, a sample was taken for universal PCR testing.

Despite this large volume of drainage and continued empiric therapy, he continued to have high‐grade fevers. As such, his antibiotics were changed to eravacycline (1 mg/kg IV every 12 h) to treat a complicated pyogenic liver abscess. However, given his history of growing up near farm animals in a potentially high‐risk setting for amebic infection (although this was many years ago), testing for *E. histolytica* was also ordered to rule out an ALA.

**FIGURE 3:**
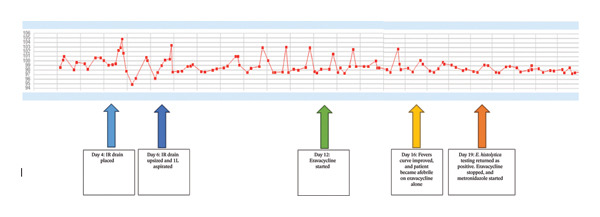
The temperature curve in degrees Fahrenheit throughout the patient’s admission and how it responded to different interventions indicated by the colored arrows. As seen above, the patient continued to have recurrent high‐grade fevers despite broad‐spectrum antibiotics and high‐volume drainage. By Day 12, all his cultures were negative despite persistent fevers. The patient was switched to eravacycline on Day 12. On eravacycline alone, the patient defervesced within 4 days of initiation and stayed afebrile before standard therapy with metronidazole was started.

Blood cultures and abscess aspirate cultures were finalized as negative. Eravacycline monotherapy was continued from Day 12 to Day 19, during which time the patient defervesced (Figure 3). Stool antigen testing returned positive for *E. histolytica*, while universal PCR for fungi and bacteria was negative. The patient was then transitioned to the standard of care, oral metronidazole. Cytology and CEA, BCG, and AFP tests from the aspirate were also negative, lowering concerns for malignancy. HIV testing was negative.

### 2.1. Treatment Outcome

Though the patient did respond to eravacycline monotherapy, this medication is not currently approved for the treatment of ALA. Hence, when *E. histolytica* stool antigen testing was positive, eravacycline was discontinued, and the patient was started on metronidazole (750 mg PO every 8 h). The patient remained afebrile. This decision was made as metronidazole is the approved standard of care for ALAs.

**FIGURE 4:**
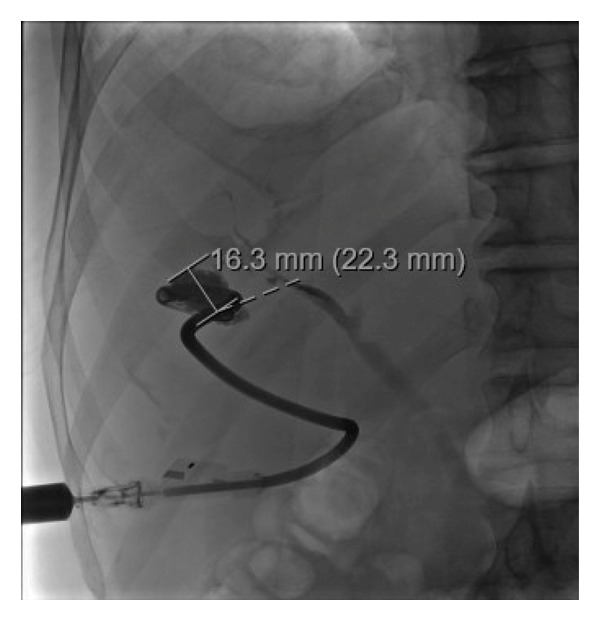
Fluoroscopic IR drain check demonstrating near total obliteration of the previously large abscess cavity before his drain was removed.

In the outpatient setting, the patient continued metronidazole therapy (750 mg PO three times per day) but then developed eosinophilia, paresthesias, and a mild rash while on treatment. Given the size of his abscess and the slow reduction in abscess size and drain output, the decision was made to continue metronidazole until complete resolution of the abscess (16 weeks total). He made an informed decision to complete his course of metronidazole for his *E. histolytica* while his symptoms were closely monitored. Additionally, he had regular outpatient fluoroscopic drain checks with IR, which showed a gradual decrease in the abscess size (see Figure 4). He also completed a course of paromomycin (750 mg PO TID for 7 days) to eradicate intraluminal cysts after metronidazole was stopped at the end of Week 16. The drain was removed after the abscess had resolved after 16 weeks of metronidazole and effective drainage. He continues to do well.

## 3. Discussion

### 3.1. Diagnostic Considerations and Differential Diagnoses

This patient is from an endemic area where he lived on farmland near livestock. This was his only identifiable risk factor for amebiasis. Initial concerns for ALA were low given his immunocompetent status, and his last known exposure being 20 years ago. Therefore, it was initially thought to be a pyogenic liver abscess. However, he was likely an asymptomatic carrier of *E. histolytica* for many years and eventually became symptomatic, such as 10% of carriers worldwide. His hyperbilirubinemia and transaminasemia were likely due to the progression and local mass effect of the enlarging abscess.

The classic “anchovy paste” appearance of his liver abscess aspirate and his positive IgG enzyme‐linked immunosorbent assay (ELISA) made an ALA more likely [4]. However, this did not confirm active infection. In patients from endemic countries, 35% of those without active infections have been found to have antiamebic antibodies from prior infection [4]. Hence, before additional testing was obtained, his treatment with broad‐spectrum antibiotics was continued.

Cultures from percutaneous drainage samples were negative, and universal PCR testing for bacteria and fungi excluded a pyogenic abscess. Cytology and tissue biopsy results were negative for malignancy. PCR testing of the abscess aspirate was unavailable at the time. Stool antigen testing was performed and returned positive (an antigen EIA test that detects only the pathogenic *E. histolytica* species and does not detect *E. dispar*). Given his clinical presentation, exposure history, radiographic features of his abscess, and classic anchovy paste appearance of the aspirate in the setting of his positive stool antigen and serum ELISA, a diagnosis of *E. histolytica* ALA was made. This was further supported by his continued improvement on metronidazole monotherapy thereafter.

### 3.2. Treatment Considerations

With proper treatment, the expected cure rate for ALAs is > 90% [4]. However, Sharma et al. [5] demonstrated in a prospective study that bilirubin > 3.5 mg/dL, encephalopathy, hypoalbuminemia (< 2.0 g/dL), and the number of abscesses were independent risk factors for increased mortality. Furthermore, neither symptom duration nor treatment type (antibiotic alone vs. antibiotics plus drainage) affected mortality [5].

The treatment of *E. histolytica* ALAs consists of a tissue agent and a luminal agent in the case of uncomplicated lesions, with the addition of percutaneous drainage for complicated lesions (left lobe lesions, lesions > 5–10 cm, and initial concern for pyogenic origin) [4]. Nitroimidazoles such as metronidazole, tinidazole, and, less commonly, ornidazole and nitazoxanide have been the mainstay of treatment since the 1960s [2, 4, 6, 7]. Metronidazole is the most commonly used and preferred therapy, with a standard duration of 7–10 days [2, 4], whereas tinidazole is an alternative agent that allows a shorter duration of therapy of 5 days [4]. In cases where patients have a slow response to metronidazole and/or drainage, as was present in this case, an extended course of metronidazole beyond the standard 7–10 days can be considered.

Luminal agents such as paramomycin and, less commonly due to limited availability, diiodohydroxyquinoline or diloxanide furoate must be used to complete therapy [4]. This is for the eradication of *E. histolytica* cysts within the intestines, which are often resistant to metronidazole and other nitroimidazoles [4]. Our patient was treated with 7 days of paromomycin to eradicate luminal cysts and prevent reinfection. This was especially important for our patient, whose hospital course was complicated by lower gastrointestinal bleeding secondary to colonic ulceration, concerning for active luminal disease.

### 3.3. Clinical Relevance of This Case and Evaluating the Utility of Newer Tetracyclines as an Alternative Effective Treatment Option

An important aspect of this case is that, while on initial empiric therapy for pyogenic liver abscess, the patient remained persistently febrile; however, defervesced shortly after initiation of eravacycline monotherapy, which is not currently a recommended guideline‐directed therapy for ALA. (see Figure 3). Currently, nitroimidazoles are the only medications recommended for the treatment of amebiasis in conjunction with luminal agents [1–4]. However, not all patients can tolerate nitroimidazoles. Given that untreated ALAs can lead to serious complications and increased mortality, and that drainage alone is ineffective [4, 8], we must consider alternative treatments when nitroimidazole therapy is contraindicated.

Seneca and Bergendahl [9] conducted a study in the 1950s, which predated the discovery of nitroimidazoles. They investigated the amebicidal effect of oxytetracycline, tetracycline, and carbomycin (a crystalline macrolide used to treat *E. histolytica* before) on cultures of 8 strains of *E. histolytica*. They compared combinations of the tetracyclines together and with carbomycin (tetracycline plus oxytetracycline vs. tetracycline plus carbomycin vs. oxytetracycline plus carbomycin) to determine which combination had the strongest synergistic inhibitory effect on the protozoa.

The study demonstrated a fourfold increase in the inhibitory effect on cultures of *E. histolytica* when equal amounts by weight of tetracycline and oxytetracycline were combined, and a more modest 2‐fold increase when either tetracycline or oxytetracycline was combined with carbomycin. Hence, the combination of tetracyclines was more effective against *E. histolytica* [9]. Furthermore, when each drug was used on its own against the 8 strains of *E. histolytica*, oxytetracycline and tetracycline were equally effective on their own, but significantly more effective than carbomycin alone. This supported the use of tetracyclines as a reasonable treatment option for *E. histolytica.*

In the early 1960s, however, metronidazole and other nitroimidazoles were discovered and found to be effective against *E. histolytica* [7, 10]. Soedin et al. [10] conducted a trial comparing the effectiveness of a 2‐g single‐dose secnidazole (a nitroimidazole‐like metronidazole) to a 5‐day course of tetracycline in combination with the antiprotozoal/antifungal clioquinol and found that there were fewer treatment failures with secnidazole compared to the tetracycline/clioquinol group. However, it is worth noting that, although clioquinol‐based regimens at this time were more cost‐effective, they were associated with more side effects [11]. Therefore, it is unclear whether the treatment failures could have been attributed to poor tolerance, especially for longer treatments. These studies led to tetracyclines falling out of favor. Eventually, metronidazole and tinidazole became and remain the standard of care for *E. histolytica* today.

Eravacycline, which was used in this case, is a novel halogenated tetracycline or “fluorocycline” which is structurally similar to tigecycline but with two additional modifications of the tetracycline core D‐ring [12]. It is approved by the FDA for the treatment of complicated intra‐abdominal pyogenic abscesses. Like other tetracyclines, it acts by binding to the 30S ribosomal subunit but has a much broader spectrum of activity than its older tetracycline cousins [12]. In fact, according to Zhanel et al. [12], eravacycline was found to be 2‐ to 4‐fold more potent against Gram‐positive cocci than tigecycline and 2‐ to 8‐fold more potent against Gram‐negative bacilli than tigecycline.

Despite multiple antibiotics and IR drainage of the liver abscess, our patient remained persistently febrile until the transition to eravacycline monotherapy (see Figure 3). Additionally, the aforementioned studies prove that combinations of older and less potent tetracyclines were effective against *E. histolytica* in the past. As such, we postulate that the significantly more potent eravacycline and potentially other halogenated tetracyclines, such as omadacycline, whether alone or in combination with other tetracyclines, may be an effective alternative regimen for *E. histolytica* infections, including ALAs, for patients who cannot tolerate nitroimidazoles. We highlight the benefit of validating this theory through randomized controlled trials comparing the efficacy of eravacycline to metronidazole to diversify treatment options for *E. histolytica* infection. Additionally, testing eravacycline’s in vitro growth‐limiting effect on *E. histolytica* axenic cultures would be a good start toward gathering more objective data on its efficacy as an alternative treatment.

### 3.4. Limitations

• The treatment course was not completed with eravacycline alone, and as such, we do not know if his fevers would have returned or how long he would need to be treated with this new drug to avoid treatment failure.
• Metronidazole is cheaper and more readily available; even if eravacycline is proven effective, its significantly higher cost would limit its use as a first‐line option. However, if it is shown to be as effective as or more effective than metronidazole, insurance companies may be inclined to cover its cost for patients with documented intolerance or allergy to metronidazole and other nitroimidazoles.
• One may argue that the patient’s drainage could confound the resolution of his fever; however, drainage occurred on Days 4 and 6, and fever did not improve until after Day 16. Therefore, this cannot account for the improvement in his fever curve with eravacycline alone before the initiation of metronidazole.

## Funding

No funding was received for this manuscript.

## Consent

No written consent has been obtained from the patient, as there are no patient‐identifiable data included in this case report.

## Conflicts of Interest

The authors declare no conflicts of interest.
